# Mapping of SARS-CoV-2 in Waldeyer’s lymphatic ring and visceral biopsies: the age and the illness duration’s impact^☆^

**DOI:** 10.1016/j.bjorl.2023.101317

**Published:** 2023-09-04

**Authors:** Jose Ramón Alba, Enrique Zapater, Cristina Martin, Dolores Ocete, Alfonso Gonzalez-Cruz, Angel Angel-de-Miguel, Carolina Ferrer, Natsuki Oishi

**Affiliations:** a University of Valencia, Faculty of Medicine and Dentistry, Valencia, Spain; b León University Hospital, ENT Department, Ponferrada, Castilla y Leon, Spain; c Valencia University General Hospital, Microbiology Department, Valencia, Spain; d Valencia University General Hospital, Radiology Department, Valencia, Spain; e Valencia University General Hospital, ENT Department, Valencia, Spain; f Valencia University General Hospital, Anesthesia Department, Valencia, Spain

**Keywords:** RT-PCR, COVID-19, Post-mortem biopsies, Aging, SARS-CoV2

## Abstract

**Objective:**

To study the impact of age and the interval between disease diagnosis and death on the organotropism of SARS-CoV-2.

**Method:**

Patients underwent post-mortem biopsies from lungs, Waldeyer ring, heart, liver, kidneys and bone marrow between 2020‒2021. SARS-CoV-2 organotropism was mapped by using molecular RT-PCR analyses for SARS-CoV2 targeting the Envelope gene (E), the RNA Polymerase Gene (RdRp), and the Nucleocapsid gene (N). Statistical and linear regression analysis was performed to study the impact of age and illness duration in SARS-CoV-2 organotropism.

**Results:**

We performed 158 postmortem biopsies in 21 patients, with a mean age of 76 years old. The mean interval between the diagnosis of the infection to the death was 23 days. The RNA of the SARS-CoV-2 was detected in 100% of lung biopsies, 76%‒82% of Waldeyer’s ring biopsies, 55% of heart biopsies, 40% of kidney biopsies, 33% of liver and 25% of bone marrow biopsies. Patients who died before the day 9, presented extensive visceral dissemination of SARS-CoV-2 RNA. Most of the patients older than 80 years (90%) presented visceral dissemination of SARS-CoV-2 RNA, while among younger patients, only 3/11 patients presented visceral dissemination of the virus. The relationship between “age” and “illness duration” and multitropism of the virus was statistically significant (*p* < 0.001).

**Conclusion:**

Disease interval and age were factors that were significantly associated with RT-PCR positive results in multiple organs. Critical COVID-19 patients have multiorganic viral dissemination in early stages. In the critical older patients, multiorganic viral dissemination is the rule.

**Level of evidence:**

4. Case Series.

## Highlights

• Disease interval and age were associated with RT-PCR positive in multiple organs.
• In older and those with a shorter disease interval: greater number of positive.
• In Waldeyer’s ring tissue and lung both factors are statistically significant.
• After 30 days, PCR+ has steel been detected in the lungs and Waldeyer’s tissue.
• RT-PCR+ in the lung and Waldeyer’s tissue, even after 65 days.

## Introduction

SARS-Cov-2 disease is an airborne disease that mainly affects the respiratory tract. Its high contagiousness is the reason that since the first case in Wuham in December 2019, it generated a worldwide pandemic in just a few months. The infection generates an uncontrolled autoimmune inflammatory response (cytokine storm) in the patient, responsible for visceral damage.^1^ Several studies presenting post-mortem findings in COVID-19 (Corona Virus Disease 2019) patients have been published^2^, ^3^, ^4^, ^5^ but very few include patients in which there was a long interval between the COVID-19 diagnosis and tissue biopsy.

Currently is known that Angiotensin-Converting Enzyme (ACE-2) which acts as a viral host cell entry receptor, is present in several organs, which is why SARS-CoV-2 can cause systemic disease with involvement of the kidneys, heart and blood vessels, liver, pancreas and the immune system.^6^ According to our search, there are no studies mapping the Reverse Transcription Polymerase Chain Reaction (RT-PCR) result of SARS-CoV-2 in different organs correlating with age and illness duration.

The aim of this study was to determine the impact of age and the interval between disease infection and death on the organotropism of SARS-CoV-2.

## Methods

This study was approved by the institutional Review Board. All cases were recruited from a single center and met the clinical diagnostic criteria for COVID-19 infection with a positive PCR test from nasopharyngeal swabs at the time of admission. With permission from the patients’ families, post-mortem biopsies were taken in the COVID-19 Intensive Care Unit (ICU) or COVID-19 Unit while wearing personal protective equipment.

Biopsies from the Waldeyer’s Ring (WR) tissue, palatine tonsil, adenoids, and posterior oropharynx mucosa were taken by an otorhinolaryngologist. The visceral organs, including the lungs, liver, kidney, bone marrow, and heart were biopsied by a radiologist using ultrasound guidance. All specimens included in this current study were obtained within 1 h of death in order to avoid post-mortem degenerative changes.

The samples were sent in physiological serum to the molecular laboratory for RT-PCR analyses for SARS-CoV2. An Allplex™ 2019-nCoV PCR assay (Seegene, Werfen) targeting the Sarbecovirus Envelope gene (E), an Ribonucleic Acid (RNA) Polymerase gene (RdRp), and SARS-CoV-2 RNA-dependent Nucleocapsid gene (N) was employed. The programe JASP (version 1.16.0.0) was used to perform the statistical and linear regression analyses.

## Results

We performed 158 postmortem biopsies in 21 patients (14 men), with a mean age of 76 years-old (range 53‒99 years). The mean interval between the illness onset to the death was 23 days (range 2‒65 days).

**Figure 1:**
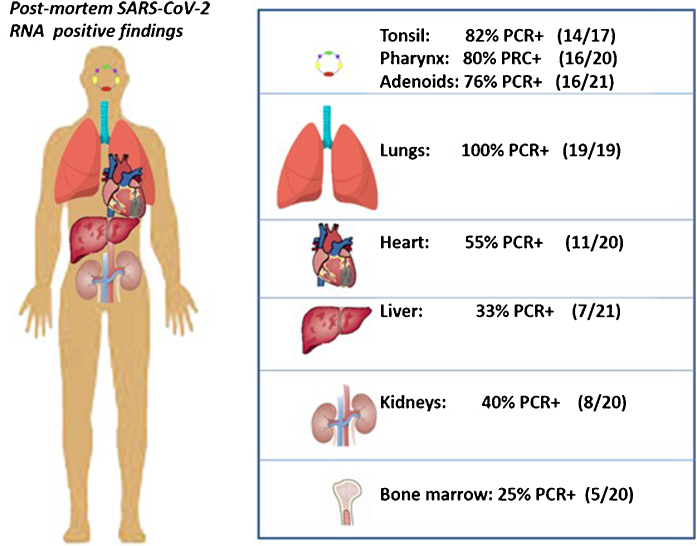
Results of 158 biopsies in 21 patients.

The RNA of the SARS-CoV-2 was detected in 100% of lung biopsies, 76%‒82% of WR biopsies, 55% of heart biopsies, 40% of kidney biopsies, 33% of liver and 25% of bone marrow biopsies (Fig. 1).

**Figure 2:**
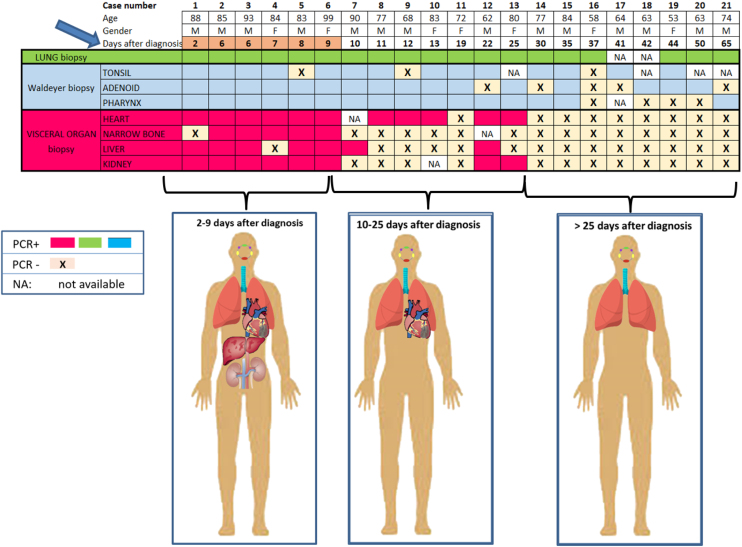
PCR+ organs depending on the day of death. An early phase is seen with viral dissemination of SARS-CoV-2 and a later phase with clearance of the virus, which persists in lung and Waldeyer’s ring.

Fig. 2 shows the results of the SARS-CoV-2 RT-PCR tests on the different patient samples; the patients are listed according to the time elapsed between the diagnosis of the infection and the biopsy. The first 6 patients included in the table, died within the first 9 days of COVID-19 diagnosis. In this short disease interval group, we detected viral RNA practically in almost all biopsies performed. From patient number 7 to 13, it is appreciated how the results of visceral biopsies tend to become negative, while lung and WR biopsies are positive. After 30 days after the infection, we only found positive PCR in the lung and WR, the rest of the biopsied visceral being negative.

**Figure 3:**
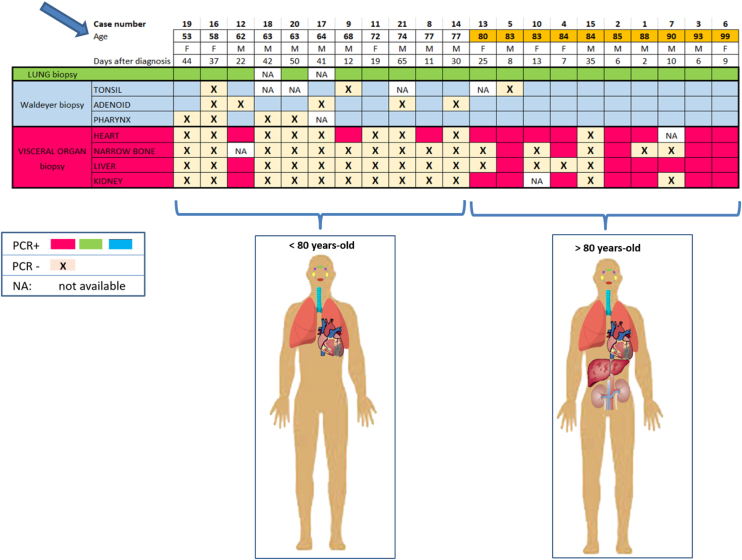
Relationship between biopsy results and patient age. The greatest visceral involvement is seen after 80 years of age.

When analyzing the age of the patient with the results of the biopsies performed, we observed that in older patients, it is when there is greater visceral involvement (Fig. 3). Visceral PCR+ was detected above all of patients aged over 80 years (90% of them). In patients under 80 years (11 patients), visceral biopsies were much less frequent, only three of them presented visceral dissemination of the virus, with the heart being the most frequently affected organ. However, all lung biopsies were positive in both age groups. Regarding those of WR, they were also positive in both groups, being more accentuated in the older group.

**Figure 4:**
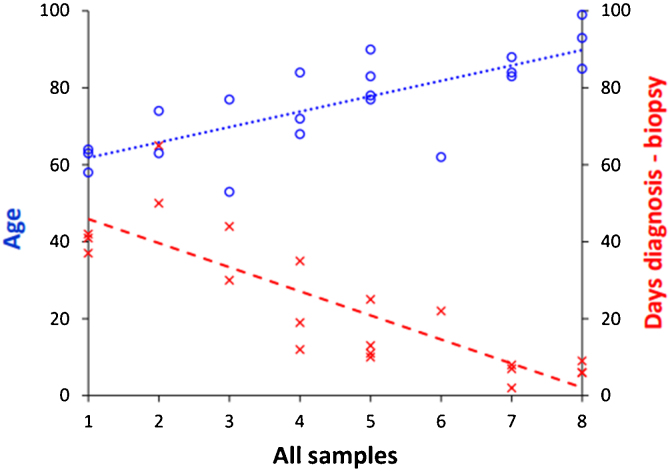
The relationship between the age and disease interval with the number of positive biopsies per patient. These variables were statistically significant such that the older the patient, the greater the number of positive tissues (*p* = 0.021), the shorter the disease interval, the greater the number of organs positive for SARS-CoV-2 detected by RT-PCR (*p* < 0.001).

The relationship between “age” and “illness duration” and multitropism of the virus was statistically significant (*p* < 0.05). The relationship between the number of positive biopsies per patient and the factors age and disease interval were statistically significant, with a greater number of positive tissues being found in older patients (*p* = 0.015) and those with a shorter disease interval (*p* = 0.002) (Fig. 4).

**Figure 5:**
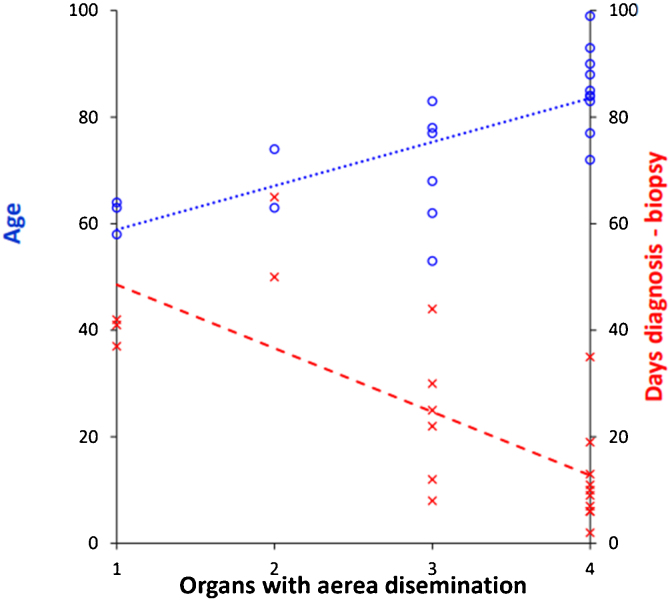
In WR tissue and lung, both factors are statistically significant: the older, and the short disease interval, the greater the dissemination aerial.

In WR tissue and lung both factors are statistically significant: the older, (t(20) = 2.20, *p* = 0.041), and the shorter interval until death, (t(20) = −2.42, *p* = 0.026), the greater the dissemination in the upper airways (Fig. 5).

In visceral organs with hematic dissemination, only the days variable is statistically significant: the fewer days elapse between the diagnosis of the infection and the biopsy, the greater the number of positive visceral organs with hematogenous dissemination (t(20) = −2.73, *p* = 0.014).

## Discussion

The early stage of SARS-CoV-2 infection is characterized by virus replication and a viremia phase, as a potential manifestation of a systemic infection.^7^ This viremia phase was reflected in our study because RT-PCR detected SARS-CoV-2 RNA widely spread throughout the body of patients who died within the first 9 days of infection, between days 10 and 25, there is less marked visceral involvement, either due to the prescribed treatment or due to the patient’s immune system. In the late phase of the infection, after 30 days, PCR+ has only been detected in the lungs and WR, confirming that it is an eminently respiratory disease.

The role of the WR system in the pathogenesis of other viral infections is well known given that microorganisms penetrate these cells and start replicating there.^8^ A similar pathway may occur in COVID-19 infections. Indeed, we observed a high percentage of positive RT-PCR results in WR tissue in the initial days of the disease because of high levels of tissue affinity and because SARS-CoV-2 virus can remain in WR tissue for a long period of time. Similar to the RT-PCR positivity results of 75% already reported,^9^ 80% positivity by RT-PCR were detected also in some of our WR samples with longer disease intervals.

It is known that the SARS-CoV-2 virus mainly affects respiratory and immune systems but that other systems are not spared, especially in elderly patients.^10^ In this current study, of the 31 RT-PCR positive results obtained in kidney, heart, bone marrow, or liver biopsies, all except five, were obtained from patients aged over 80 years. Indeed, age was a statistically significant factor: the older the patient, the greater the number of positive tissues identified independently from duration of the disease. Aging itself drives a loss of immune system function, including dysregulation of innate immunity and altered T-cell and B-cell maturation and differentiation.^11^ A weakened immune system would explain why visceral involvement is more frequent in elderly patients as well as the long-term survival of SARS-CoV-2 in these immunocompromised patients.^12^

We consider that both age of the patient and disease interval are determining factors in the viral detection at the different organs studied being the disease interval more significative than the age regarding systemic infection. The viability of this virus after a prolonged interval remains unknown.

## Conclusion

Disease interval and age were factors that were significantly associated with RT-PCR positive results in multiple organs. Using RT-PCR, the presence SARS-CoV-2 RNA was widely spread throughout the bodies of patients who died after a short COVID-19 disease interval. In the group of patients who died later than 25 days after diagnosis of infection, the RT-PCR detected SARS-CoV-2 only in the lung and WR tissue, even after intervals of up to 65 days.

## Justification for 8 authors

This is a multidisciplinary study, many specialists have participated in the design, methodology, taking biopsies from patients, processing, data analysis, and conclusions.

We have selected 8 of all the contributors to the paper, I kindly ask you to accept the authorship.

## Funding

None.

Data sharing is not applicable to this article as no new data were created or analyzed in this study.

## Conflicts of interest

The authors declare no conflicts of interest.
